# Laparoscopic treatment of giant ovarian cystic tumors in children: Case report

**DOI:** 10.1097/MD.0000000000042920

**Published:** 2025-06-20

**Authors:** Huashan Zhao, Shumin Zhao, Shisong Zhang, Rui Guo, Hongxiu Xu, Sai Huang, Gang Shen, Yunpeng Zhai

**Affiliations:** a Department of Thoracic and Oncological Surgery, Children’s Hospital Affiliated to Shandong University, Jinan, China; b Department of Thoracic and Oncological Surgery, Jinan Children’s Hospital, Jinan, China; c The Affiliated Central Hospital of Shandong First Medical University (Jinan Central Hospital), Jinan, China.

**Keywords:** children, laparoscopic surgery, ovarian cyst, ovarian cystic tumors, ovarian lesions

## Abstract

**Rationale:**

Ovarian cystic tumors are common in adult women. However, cases of ovarian cystic tumors approximately 30 cm in diameter are rarely reported in adult women and even rarer in children. Here, we report a rare case of a giant ovarian cystic tumor in a 13-year-old girl.

**Patient concerns:**

A 13-year-old girl was admitted to hospital with abdominal pain and distension for 7 days. The parents felt anxious about the child’s abdominal lesions and requested laparoscopic surgery.

**Diagnoses:**

After evaluation at our hospital, abdominal color ultrasound and enhanced computer tomography revealed a large sac-like fluid density shadow with clear edges in the abdomen, approximately 38 cm × 35 cm × 24 cm in size. We performed a laparoscopic resection of the cystic ovarian tumor, which was pathologically confirmed as a benign ovarian tumor.

**Interventions:**

Subsequently, laparoscopic exploration revealed a huge cystic tumor originated from the right ovary with a clear boundary between the tumor and the right ovary.

**Outcomes:**

Laparoscopic surgery was used to remove the lesion, and the postoperative recovery and follow-up were uneventful.

**Lessons:**

Cases of ovarian cystic tumors with a diameter of approximately 30 cm are very rare in pediatric patients. Laparoscopic surgery is an essential method for the diagnosis and treatment of giant ovarian cystic tumors and can be used as a first-line treatment.

## 1. Introduction

Ovarian cysts are approximately 14% more common in women of reproductive age^[1]^ but are significantly less common in girls than in adults.^[2,3]^ Some ovarian cysts in childhood are small and biogenic and may resolve spontaneously.^[4]^ Most early ovarian cysts have no obvious clinical symptoms, and when the cyst gradually grows, the patient may have abdominal discomfort, abnormal menstruation, ovarian torsion, infection, bleeding, and other secondary manifestations.^[4–7]^ This report describes a 13-year-old girl, one of twins, who developed a large cystic tumor on the right ovary, which mainly manifested with abdominal discomfort. We successfully performed laparoscopic surgery to remove the large cystic tumor on the right ovary and the cystic tumor on the left ovary. The ovaries were repaired. This report aims to improve the understanding of giant ovarian cystic tumors in children and reduce misdiagnosis and missed diagnosis. Laparoscopic surgery has great advantages for the treatment of giant ovarian cystic tumors and can be used as the 1st choice of procedure. This case report meets SCARE criteria,^[8]^ and informed written consent was obtained from the patient’s family.

## 2. Case description

### 2.1. General information

The patient weighed 124 kg and was 153 cm tall. Vital signs included a heart rate of 121 beats/min, breathing 25 minutes, and blood pressure 141/82 mm Hg. Routine blood tests, including liver, kidney, and coagulation functions were within the normal range. She was admitted with abdominal pain with bloating for 7 days. The abdominal pain was tolerable, with no nausea, vomiting, and her temperature was normal. The parents observed that the abdominal distention of the patient was more evident than that of her twin sister; she had poor exercise tolerance and tired easily. Abdominal computer tomography (CT) was performed and detected a large cystic tumor. The patient had regular menstruation with no obvious abnormality. Her menstruation ended 2 days before admission. There was no family history of any related genetic disease. Abdominal and pelvic ultrasonography of the twin sister showed no abnormalities. The parents felt anxious about the child’s abdominal lesions and requested laparoscopic surgery. After hospitalization, the patient underwent all relevant examinations and tests, and she recovered normally.

### 2.2. Additional examinations

**Figure 1.**
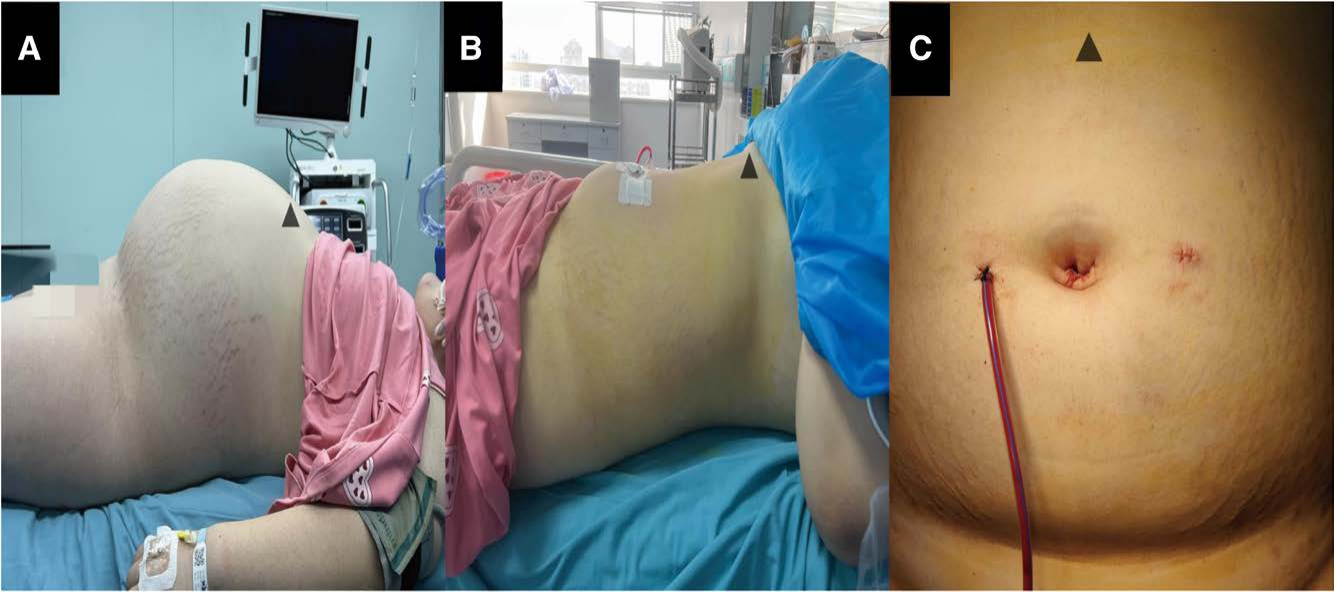
Figure (A) shows the abdominal distention preoperatively, with the triangle representing the head. Figure (B) represents the disappearance of abdominal distension postoperatively, with the triangle representing the head. Figure (C) shows the incision appearance postoperatively, with the triangle representing the head.

**Figure 2.**
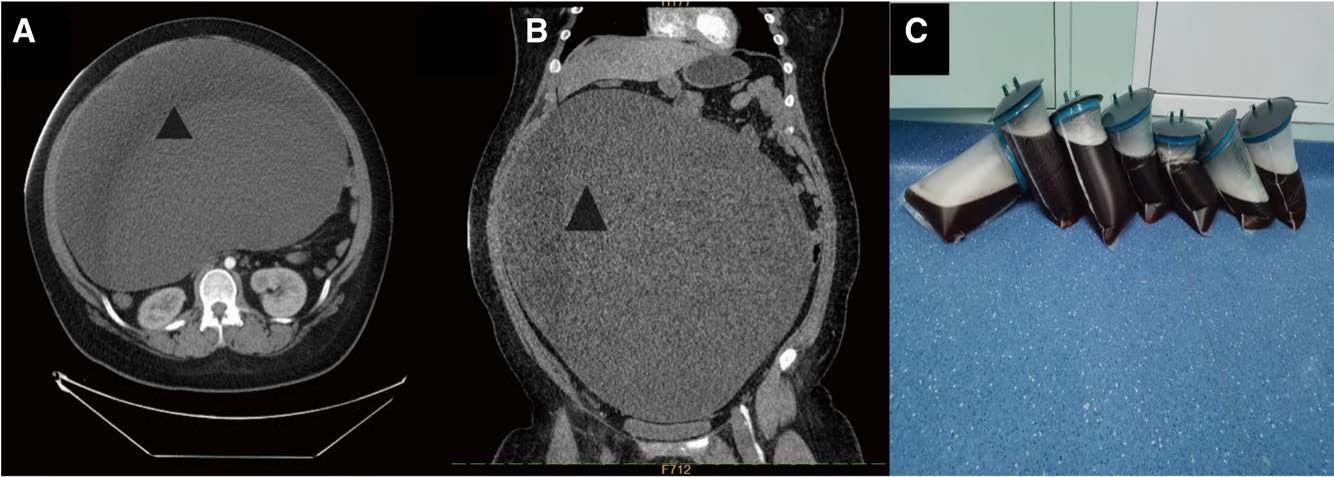
(A and B) Abdominal enhanced CT showing large abdominal tumors with triangles. Figure (C) is the sac fluid extracted from the giant ovarian cystic tumor intraoperatively. CT = computer tomography.

On physical examination, the patient’s abdomen was distended, a mass measuring approximately 30 cm × 25 cm × 25 cm was palpable, with limited motion and occupying the entire abdomen (Fig. 1A). The patient had no tenderness, rebound pain, or muscle tension in the abdomen. Enhanced abdominal CT showed abdominal distention, a large sac-like fluid density shadow with clear edges, approximately 38 cm × 35 cm × 24 cm in size, with uneven internal density. No abnormality was observed. The lesion was extensive, and the surrounding organs were under obvious pressure (Fig. 2A,B). Brain and chest examination revealed no abnormality. Alpha-fetoprotein, human chorionic gonadotropin, cancer antigen 125, and estrogen levels were in the normal range.

### 2.3. Surgical procedure

**Figure 3.**
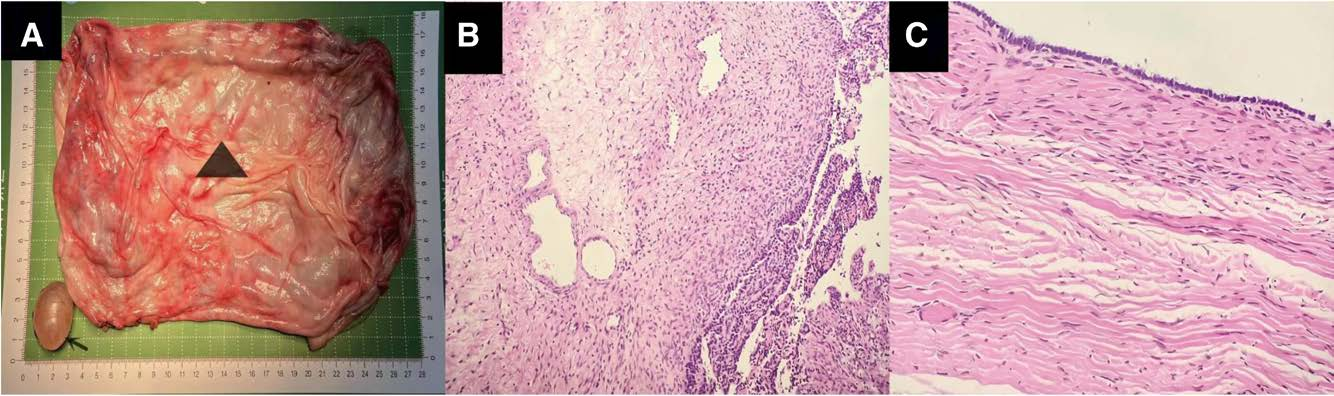
(A) The triangle is the giant cystic tumor of the right ovary specimen, and the arrow is the cyst of the left ovary specimen. Figure (B) shows HE staining of the follicular cyst the right ovarian tumor, with pathological images of ×100, most of the lining epithelial cells of the cyst wall were shed, and the fibrous tissue of the cyst wall was hyperplastic. Figure (C) shows an accessory mesonephric duct of the ovary, HE staining of the left ovarian tumor showed that the cyst wall had single cubic epithelium in the pathological image of ×100. HE = hematoxylin and eosin.

Based on history, physical examination, imaging, and laboratory examination, the possibility of ovarian cystic tumor was considered. Due to the large size of the tumor and its mainly cystic component, we decided to conduct a laparoscopic surgical exploration. Under general anesthesia, the patient was placed in a supine position. A 10 mm incision was made at the lower umbilical margin, and the incision was made into the abdomen by layer. A puncture needle was placed under direct vision to extract the fluid in the cystic tumor slowly, and approximately 13,000 mL was extracted (Fig. 2C). The fluid extraction process was slow; the blood pressure and heart rate were stable. Then, a 10 mm trocar was inserted into the subumbilical incision to establish CO_2_ pneumoperitoneum; abdominal pressure was 8 mm Hg, and flow was 6 L/min. After the trocar was placed into the laparoscope for observation, a 5 mm incision was made on the left and right middle abdomen to insert a 5 mm trocar. The cystic tumor originated from the right ovary, and the boundary between the tumor and the right ovary was clear. Additionally, the left ovary had a 3.5 cm × 2.5 cm cyst. After informing the parents about the condition during the surgery, the parents requested removal of the left ovarian cyst. We 1st treated the huge cystic tumor of the right ovary. The tumor was completely removed along the boundary between the tumor and the right ovary. The right ovary was reconstructed using a 4-0 absorbable suture, then the left ovarian cyst was completely removed. The left ovary was reconstructed using a 4-0 absorbable suture. An extract bag was used to remove the diseased tissue in its entirety (Fig. 3A). An indwelling pelvic drainage tube was placed. No bleeding was detected, and the incisions were sutured layer by layer. Figure 1C shows the incision after the laparoscopic surgery, with the triangle representing the head end. The operation was successfully completed, and the abdominal distension disappeared. Figure 1B shows that the obvious abdominal distension disappeared after surgery. The patient resumed her regular diet 2 days postoperatively. The pelvic drainage tube was removed 4 days postoperatively, and the patient was discharged 5 days postoperatively. Abdominal ultrasound examinations 3, 6, and 12 months postoperatively found no abnormality.

### 2.4. Pathological findings

Figure 3A – the triangle is the giant cystic tumor of the right ovary specimen, and the arrow is the cyst of the left ovary specimen. Figure 3B shows the cystic tumor of the right ovary was benign. Hematoxylin and eosin staining showed that most of the epithelial cells lining the cyst wall were shed, and fibrous tissue proliferated in the cyst wall. The left ovarian tumor was characterized by a single cubic epithelium on the hematoxylin and eosin-stained ×100 pathological picture (Fig. 3C).

### 2.5. Nursing roadmap

**Figure 4.**
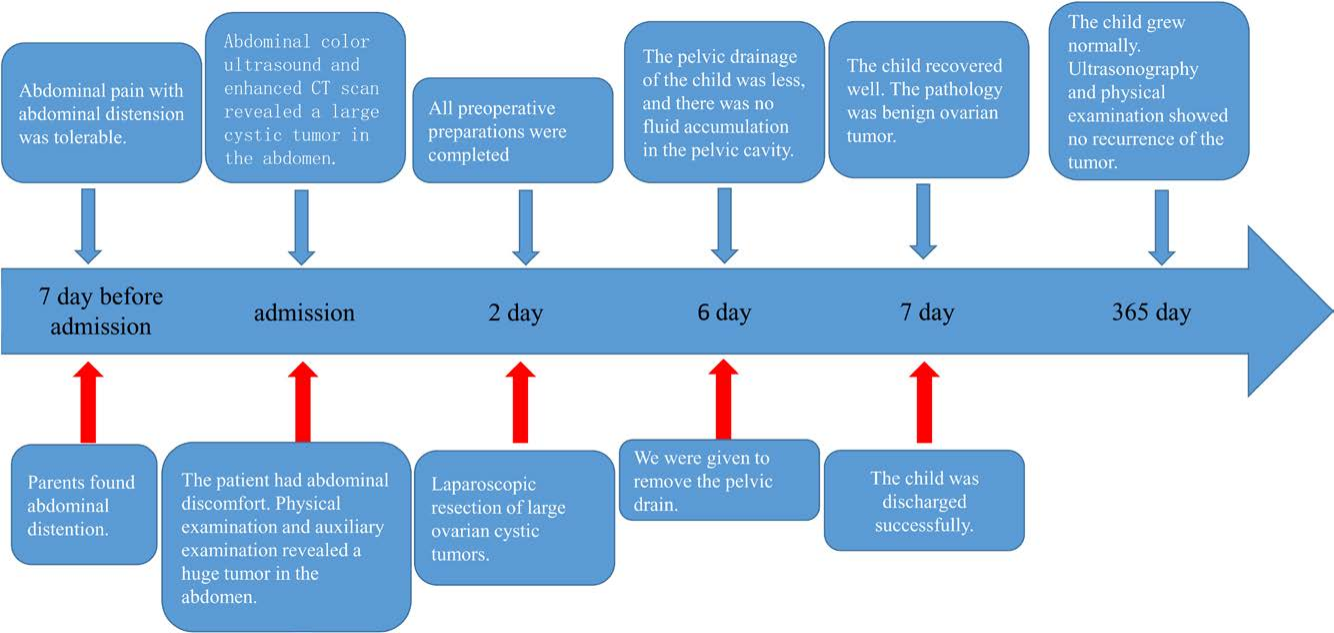
Nursing roadmap. CT = computer tomography.

The nursing roadmap in this paper is shown in Figure 4.

## 3. Discussion

Among ovarian cysts, giant ovarian cysts in children are rare. The specific cause of ovarian cysts is unclear; however, it is believed to be related to environment, diet, endocrine, heredity, and so on.^[9–12]^ Giant ovarian cysts with a diameter of approximately 30 cm are rarely reported in adults^[1]^ and even rarer in children.^[13]^ Most ovarian cysts have no clinical symptoms; however, when the cyst ruptures, abdominal pain may occur, which can be severe in some cases. When a huge ovarian cyst causes substantial pressure on the surrounding areas, non-characteristic symptoms such as abdominal discomfort, poor diet, and poor mental condition can occur.^[1]^ Shresth et al^[1]^ reported a 32-year-old woman with a rare giant ovarian cystic tumor with clinical manifestations of abdominal distension and progressive breathing and walking difficulties. In our case, due to the huge ovarian cystic tumor, there were obvious symptoms of pressure on the surrounding organs, including abdominal discomfort, poor diet, poor mental condition, and substantially decreased endurance compared with that of the twin sister. It is worth noting that most giant ovarian cystic tumors reported in the previous literature were adults, while in this case, the symptoms of a giant ovarian cystic tumor appeared at a younger age.

Ovarian cysts can be divided into functional, neoplastic, endometritic, and tubal. Functional cysts are relatively rare in children, mainly luteal and follicular cysts, and most resolve spontaneously under observation or drug intervention.^[14]^ The incidence of neoplastic cysts, including teratoma, dermoid cysts, and so on, is higher in children. Benign tumors can account for more than 95%.^[15]^ Endometriosis cysts, also known as chocolate cysts, are rare in children.^[16]^ Tubal and ovarian cysts are mostly located on the mesangium of the ovaries and fallopian tubes. They are chronic pelvic inflammatory disease lesions and are not true ovarian cysts.^[17]^ Therefore, most pediatric ovarian cysts are benign, and only a few are malignant. In our case report, pathology revealed hyperplasia of epithelial tissue and fibrous tissue, which were diagnosed as benign ovarian tumors.

Ovarian cysts have no specific symptoms. A few are found due to torsion, rupture, and acute abdomen, most are inadvertently found during physical examination. Mesenteric and retroperitoneal cystic tumors should also be considered in the differential diagnosis of large cystic tumors in the abdominal cavity of children.^[18]^ Ultrasonography is the most commonly used method to detect ovarian cysts, with high accuracy.^[19]^ When the ultrasonic features of ovarian cysts are atypical, or the lesions continue to grow, magnetic resonance imaging can improve the diagnosis specificity and distinguish teratoma, endometriosis cyst, ovarian fibroma, and so on.^[20]^ Enhanced CT can be used to identify the site of the primary tumor and assist in the identification of tumor metastases.^[21]^ In our case, the tumor was large. Ultrasound and enhanced CT determined a low risk of ovarian malignancy. Unfortunately, the patient declined the magnetic resonance imaging because of its high cost. Laparoscopic surgery can be of great value in understanding the source of the lesion and the relationship of structures around the lesion, and can be used to remove the lesion at the same time as the lesion is detected.^[22]^

Ovarian cyst treatment can be divided into conservative and surgical treatments. Most ovarian cysts are treated conservatively with observation. Ovarian cysts: commonly have a diameter >5 cm, tend to be malignant, and ovarian cyst torsion and other symptoms require surgical treatment.^[23,24]^ At present, most surgical treatments are laparoscopic. However, in our case, the tumor was approximately 30 cm in diameter and occupied the entire abdominal and pelvic cavity. Surgery was challenging. Preoperatively, we ruled out the possibility of malignancy. Great care was taken to protect the surrounding tissues and organs intraoperatively. Laparoscopy was used to remove the fluid in the cyst directly, and then the ovarian cyst was completely resected, and the ovaries were repaired. Postoperative pathology showed benign ovarian cysts. Laparoscopic surgery has great advantages for children, with less damage, less scarring, and faster recovery. This is why we insisted on laparoscopic surgery in this case.

## 4. Conclusions

Cases of giant ovarian cystic tumors approximately 30 cm in diameter in children are rare. Complete laparoscopic resection of the tumor was successful. The patient recovered smoothly after surgery, and no recurrence was observed during 1-year follow-up. We hope our report provides clinicians with ideas for diagnosis and treatment to provide better medical care to children.

## Acknowledgments

We would like to thank Editage (www.editage.cn) for English language editing.

## Author contributions

**Conceptualization:** Huashan Zhao, Shisong Zhang, Rui Guo.

**Data curation:** Huashan Zhao, Shumin Zhao, Shisong Zhang, Rui Guo, Hongxiu Xu, Gang Shen.

**Formal analysis:** Huashan Zhao, Gang Shen.

**Investigation:** Shumin Zhao, Shisong Zhang, Hongxiu Xu.

**Methodology:** Shumin Zhao, Hongxiu Xu, Sai Huang.

**Software:** Hongxiu Xu.

**Supervision:** Sai Huang.

**Writing – original draft:** Huashan Zhao, Yunpeng Zhai.

**Writing – review & editing:** Huashan Zhao, Yunpeng Zhai.
